# Supplementary material for: Mining the UniProtKB/Swiss‐Prot database for antimicrobial peptides
Source: Protein Sci. 2025 Mar 18;34(4):e70083. doi: 10.1002/pro.70083 (PMC11917140; doi:10.1002/pro.70083)
Supplement: Supplementary file 1 — Data S1. Supporting Information. [file PRO-34-e70083-s001.pdf]

Supporting Information

Mining the UniProtKB/Swiss-Prot database for antimicrobial peptides

Chenkai Li[1, 2], Darcy Sutherland[1, 3, 4], Ali Salehi[1, 3], Amelia Richter[1, 3], Diana Lin[1], Sambina Islam Aninta[1], Hossein Ebrahimikondori[1, 2], Anat Yanai[1, 3], Lauren Coombe[1], René L. Warren[1], Monica Kotkoff[1], Linda M.N. Hoang[3, 4], Caren C. Helbing[5], and Inanc Birol[1, 3, 4, 6 *]

1. Canada's Michael Smith Genome Sciences Centre, BC Cancer Agency, Vancouver, BC, V5Z 4S6, Canada
2. Bioinformatics Graduate Program, University of British Columbia, Vancouver, BC, V6T 1Z4, Canada
3. Public Health Laboratory, British Columbia Centre for Disease Control, Vancouver, BC, V5Z 4R4, Canada
4. Department of Pathology and Laboratory Medicine, University of British Columbia, Vancouver, BC, V6T 1Z4, Canada
5. Department of Biochemistry and Microbiology, University of Victoria, Victoria, BC, V8P 5C2, Canada
6. Department of Medical Genetics, University of British Columbia, Vancouver, BC, V6H 3N1, Canada

* Correspondence: Inanc Birol (ibirol@bcgsc.ca)

Supplementary Figures

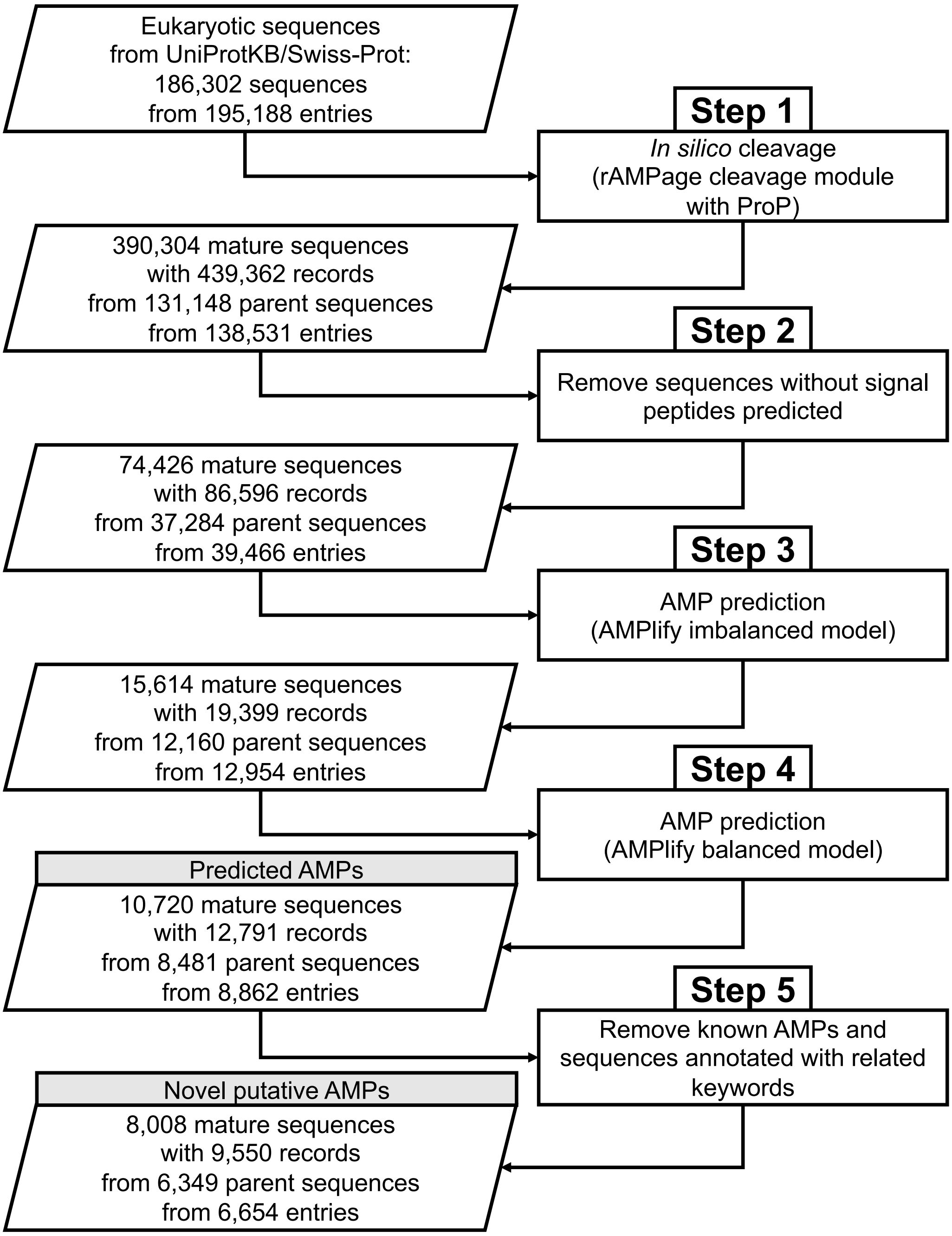

Figure S1: AMP mining workflow. The AMP mining workflow utilizes the rAMPage (Lin et al. 2022) cleavage module with ProP (Duckert et al. 2004) to cleave putative precursor sequences, and AMPlify (Li et al. 2022; Li et al. 2023) to predict AMP sequences. The numbers of candidate mature peptide sequences, candidate mature peptide sequence records, parent sequences, and UniProt entries, that remained at each step are reported. We note that two candidate mature peptide sequence records are considered distinct from each other if any of these three attributes are different: UniProt entry ID, position in the parent sequence, and candidate mature peptide sequence. All numbers presented in the workflow are non-redundant.

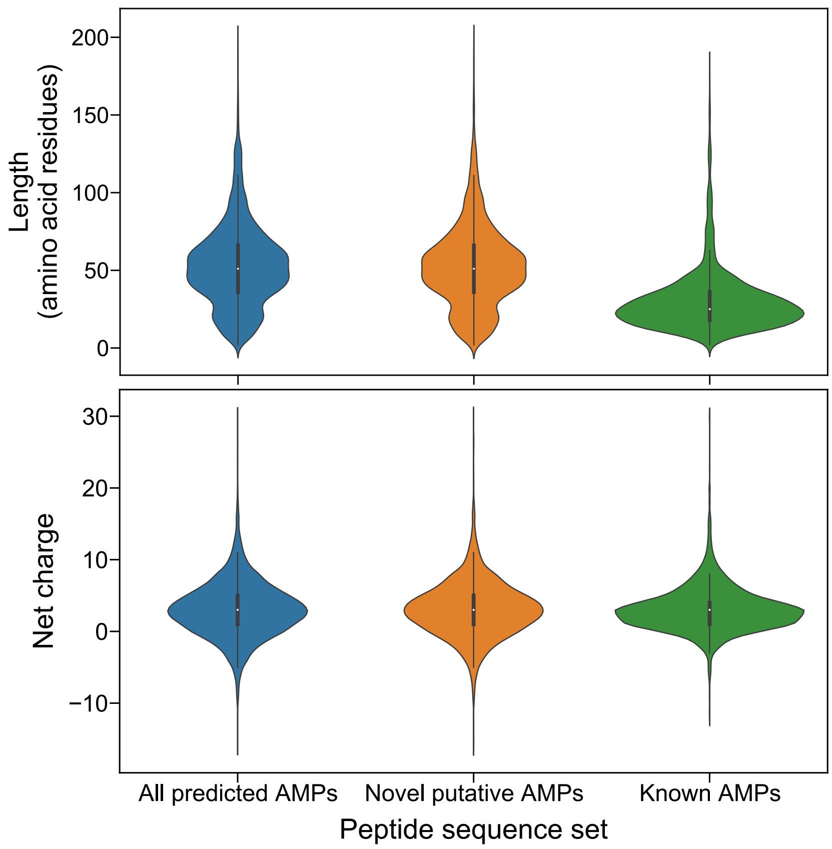

Figure S2: Length and net charge distributions of the AMPs predicted by AMPlify from the UniProtKB/Swiss-Prot database. Length and net charge distributions were calculated for all 10,720 predicted AMPs as well as the 8,008 novel putative AMPs among them. The distributions of the 4,538 known AMP sequences from Antimicrobial Peptide Database (APD3) (Wang et al. 2016) and Database of Anuran Defense Peptides (DADP) (Novković et al. 2012) were plotted alongside for comparison. Mean (μ) and standard deviation (σ) values of each distribution are as follows: all predicted AMPs (length: μ = 52.83 aa, σ = 26.59 aa; net charge: μ= 3.02, σ = 3.90), novel putative AMPs (length: μ = 52.49 aa, σ = 26.49 aa; net charge: μ = 3.04, σ = 3.88), and known AMPs (length: μ = 30.21 aa, σ = 20.28 aa; net charge: μ = 3.05, σ = 3.10).

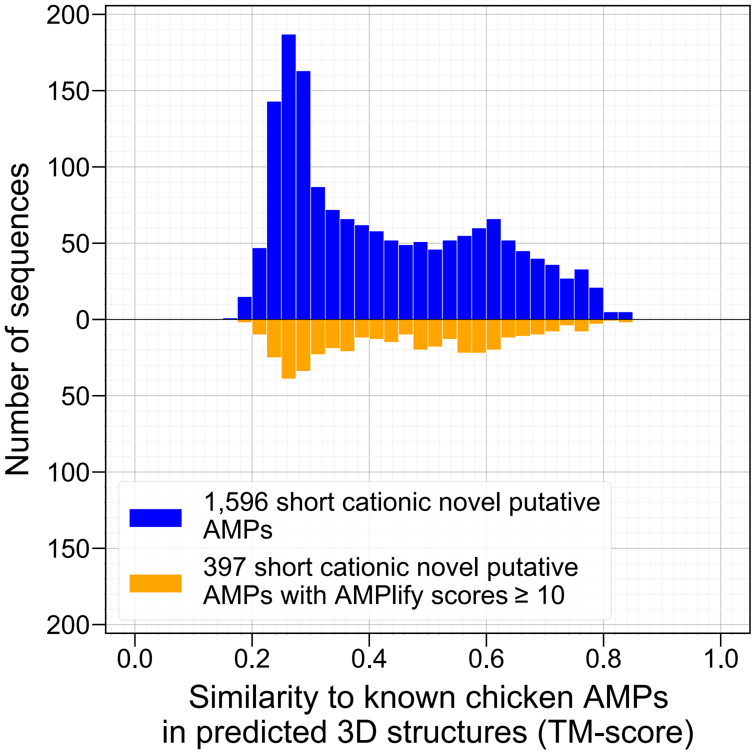

Figure S3: Similarity distributions of the short cationic novel putative AMPs mined from the UniProtKB/Swiss-Prot database to known chicken AMPs in predicted three-dimensional structures. The similarity distribution of 1,596 short cationic novel putative AMPs to known chicken AMPs in predicted three-dimensional (3D) structures holds a mean of 0.4266 and a standard deviation of 0.1690. Among all short cationic novel putative AMPs, 397 of them are with AMPlify scores ≥ 10 (i.e., AMPlify probability scores ≥ 0.9). The similarity distribution of these 397 putative AMPs to known chicken AMPs in predicted 3D structures holds a mean of 0.4434 and a standard deviation of 0.1611. The similarity of each putative AMP to known chicken AMPs in predicted 3D structures (TM-score) was considered as the similarity of the putative AMP to the most similar known chicken AMP in predicted 3D structures (i.e., reference chicken AMP) from Antimicrobial Peptide Database (APD3) (Wang et al. 2016), based on which the distributions were plotted.

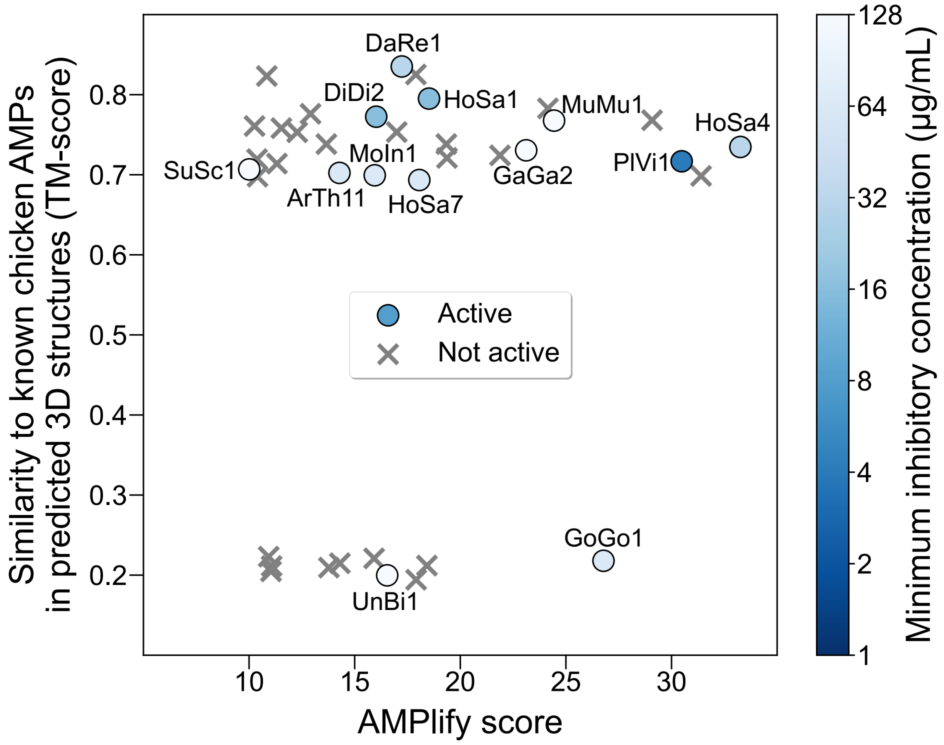

Figure S4: Visualization of antimicrobial activity of the 38 tested putative AMPs with respect to AMPlify scores and associated similarities to known chicken AMPs in predicted three-dimensional structures. Peptides without any observable antimicrobial activity are presented as grey crosses, and the active peptides are presented in blue dots with their names annotated. Dots with darker colors indicate stronger antimicrobial activity against *Escherichia coli* ATCC 25922, determined by the lowest minimum inhibitory concentration (MIC) value of each peptide against the strain. AMPlify scores from the balanced model were used for visualization. The similarity of each tested peptide to known chicken AMPs in predicted three-dimensional (3D) structures (TM-score) was considered as the similarity of that peptide to the most similar known chicken AMP in predicted 3D structures (i.e., reference chicken AMP) from Antimicrobial Peptide Database (APD3) (Wang et al. 2016).

Supplementary Tables

Table S1: Performance comparison among different tools on the balanced test set. Values of accuracy (acc), sensitivity (sens), specificity (spec), F1 score (F1) and area under the receiver operating characteristic curve (AUROC) are presented in percentages.

Tool	Model	Acc	Sens	Spec	F1	AUROC
iAMPpred (Meher et al. 2017)	original[a]	74.01	87.90	60.12	77.18	80.70
iAMP-2L (Xiao et al. 2013)	original[a]	77.96	88.26	67.66	80.02	—*
AMP Scanner Vr.2 (Veltri et al. 2018)	original[a]	78.50	90.66	66.35	80.83	88.33
AMPlify (Li et al. 2022; Li et al. 2023)	balanced	**93.71**	92.93	94.49	**93.66**	**98.37**
	imbalanced	89.22	**94.37**	84.07	89.75	96.88
	balanced + imbalanced[b]	93.59	91.50	**95.69**	93.46	—*

[a] Models presented in the referenced papers, which are available through online servers.

[b] Only sequences predicted as AMPs by both models are determined to be AMPs.

* AUROC values were not calculated for models which only output predicted labels instead of probabilities.

Table S2: Performance comparison among different tools on the imbalanced test set. Values of accuracy (acc), sensitivity (sens), specificity (spec), F1 score (F1) and area under the receiver operating characteristic curve (AUROC) are presented in percentages. We note that the online server of iAMP-2L (Xiao et al. 2013) was down at the time this analysis was done (November 11, 2022), so its results were not compared here.

Tool	Model	Acc	Sens	Spec	F1	AUROC
iAMPpred (Meher et al. 2017)	original[a]	14.29	87.90	11.90	6.07	55.54
AMP Scanner Vr.2 (Veltri et al. 2018)	original[a]	71.26	90.66	70.63	16.57	91.44
AMPlify (Li et al. 2022; Li et al. 2023)	balanced	95.97	92.93	96.06	59.19	98.06
	imbalanced	98.94	**94.37**	99.09	84.87	**99.54**
	balanced + imbalanced[b]	**99.31**	91.50	**99.56**	**89.25**	—[*]

[a] Models presented in the referenced papers, which are available through online servers.

[b] Only sequences predicted as AMPs by both models are determined to be AMPs.

[*] AUROC values were not calculated for models which only output predicted labels instead of probabilities.

Table S3: Characteristics of the 40 putative AMP sequences mined from the UniProtKB/Swiss-Prot database that have been prioritized for synthesis. A total number of 397 short cationic novel putative AMPs with AMPlify scores ≥ 10 (i.e., AMPlify probability scores ≥ 0.9) were compared with known chicken AMPs for their similarities in predicted three-dimensional (3D) structures. The top section shows the 30 most similar putative AMPs to the known chicken AMPs in predicted 3D structures as measured by TM-scores, while the bottom section shows the 10 least similar ones to the known chicken AMPs in predicted 3D structures.

Peptide name	Sequence	# aa	Net charge[a]	Molecular weight (Da)	AMPlify score[b]	Sequence similarity to known AMPs[c] (%)	Similarity to known chicken AMPs in predicted 3D structures[d] (TM-score)	Reference chicken AMP[e]
DaRe1	WLLVLQRGHRLASIKHVCQ LSERKR	25	5	3027.63	17.23	40.00	0.8352	Chicken CATH-2
ScPo1	AWDWAKNWVFWTCSYLL NFLYHHHCSRDLIRR	32	2	4138.74	17.90	37.50	0.8252	Chicken CATH-2
ArTh10	DLWFGLKTEGELAFVKRTI FDVIYKSAKRKR	31	4	3716.39	10.84	35.48	0.8233	Chicken CATH-2
HoSa1	GLQKLINKIKSQMSRFSTKT NKICGP	26	6	2921.51	18.52	38.46	0.7951	Chicken CATH-2
HoSa2	TRWEAMKAKATELRVCCA RRKR	22	6	2664.21	24.15	33.33	0.7829	Chicken CATH-3
DiVi1	VWEFFEAGAGLRASNASK KIYGVAKRFRR	29	5	3315.83	12.91	36.36	0.7761	Chicken CATH-3
DiDi2	FWNLNKKKKFFYKTVKNSI GQVILRDMSNN	30	6	3662.31	16.02	33.33	0.7725	Chicken CATH-2
DiDi3*	GVLKIPLAFVQMIAISIALIC	25	1	2650.45	30.78	44.00	0.7724	Chicken

	LLIP							CATH-3
LySt2	FMRFGKRFMRFGRFGKSA EVENNIQIAAKQS	31	5	3652.26	29.08	35.48	0.7681	Chicken CATH-3
MuMu1	RWSQWAYAGRAQFCAVRR SVFGFSVRSGMVCRPRR	35	8	4135.81	24.44	34.29	0.7676	Chicken CATH-2
CaGl1	SYWWWGFHKNVIDRREAF YADLAEKKKAEN	30	1	3759.20	10.26	33.33	0.7608	Chicken CATH-3
HoSa3	AKETLEKKKLLKELWESSK KVH	22	3	2653.16	11.53	40.91	0.7582	Chicken CATH-3
BoMo2	AWSSLHSGWAKRAWQDM SSAWGKR	24	3	2790.11	16.99	33.33	0.7533	Chicken CATH-2
ScPo2	FMIHNTLGLFYRSVVRNEI KKR	22	4	2722.25	12.28	36.36	0.7531	Chicken CATH-3
DiDi1	FTLFFPIFMIVVCVISFFNLH KR	23	2	2819.51	19.34	34.78	0.7383	Chicken CATH-3
RaNo1	YFAFYNLFHCLKKDSNNVE MYLKLLKCRLIRSKC	34	5	4203.07	13.66	35.29	0.7383	Chicken CATH-3
HoSa4	WIIQCWWRQVLEKLLAKR RR	20	5	2682.28	33.26	35.00	0.7347	Chicken CATH-3
GaGa2	GLIKRIIRQKR	11	5	1380.75	23.12	45.45	0.7305	Ovipin
MuMu2*	ILTPVSTVLALLLIAALILLK R	22	2	2345.04	10.44	40.91	0.7250	Chicken CATH-3
RaNo2	ASWEYLVHHVMAMGAFFS GIFWKR	24	1	2871.37	21.88	29.17	0.7242	Chicken CATH-2
HoSa5	HTSRLKARKHSKRRVRYIC	25	7	3111.67	19.37	32.00	0.7210	Chicken

	EFTIPQ							CATH-3
EiHe1	ALWKLGEPSDHLLQWLVL HLASLHLRLLFKR	31	2	3704.47	10.38	38.71	0.7195	Chicken CATH-3
PlVi1	ILDKVRLWLARIRLNLLKR	19	5	2390.01	30.48	40.00	0.7168	Chicken CATH-3
MuMu3	YFAFHNLFHCLKKDSSHVE MYLKLLKCRLIQSNC	34	3	4130.92	11.32	32.35	0.7139	Chicken CATH-2
SuSc1	ARLDVAAEFRKKWNKWAL SRGKR	23	6	2787.27	10.01	34.78	0.7067	Chicken CATH-3
ArTh11	RSIIRNGIRTLTWRKETKRK KK	22	9	2769.34	14.28	36.36	0.7023	Chicken CATH-3
MoIn1	VLKKSEIRIDFISRTILHI	19	2	2281.77	15.95	31.58	0.6993	Chicken CATH-2
HoSa6	GVLKKVIRHKR	11	5	1333.69	31.40	42.11	0.6985	Ovipin
CaGl2	QFFLPMILRLYVSRLFISKL	20	3	2485.11	10.40	50.00	0.6976	Chicken CATH-3
HoSa7	LYVFCCRTRAKTPSVIYTIN LVVTDLLVGLSLPTR	35	3	3926.73	18.06	28.57	0.6934	Chicken CATH-3
GaGa4	ARSKKSPWPGWAPLAAPH SH	20	3	2181.49	10.93	35.00	0.2230	OvoDBβ
OrSa6	VMRSAGSRSSKR	12	4	1321.52	15.92	37.50	0.2209	OvoDBβ
GoGo1	GRGRPKGRGRGRPRGRPR GSKR	22	11	2457.84	26.78	44.12	0.2179	CHB1
HoSa8	QVWKRR	6	3	872.04	14.30	42.86	0.2148	Ovipin
HoSa9	FQWHGRKPGPETGVPQSRP	23	3	2624.99	18.42	34.78	0.2119	CHB2

	PIPR							
DrMe6	RIRGHTIKG	9	3	1037.23	11.07	37.50	0.2113	Chicken Heterophil Peptide 2
MuMu4	FLEKPPGPLGARPLGGK	17	2	1734.07	13.78	47.06	0.2094	OvoDBβ
HoSa10	TPKFVGK	7	2	775.95	11.04	41.67	0.2047	OvoDBβ
UnBi1	VRCRFACC	8	2	957.19	16.54	41.67	0.1997	Ovipin
ScPo3	SPLHKR	6	2	736.87	17.91	42.86	0.1942	Ovipin

[a] Net charge at pH = 7.

[b] AMPlify scores from the balanced model. AMPlify scores range from 0 to 80; sequences with AMPlify scores > 3.01 (i.e., AMPlify probability scores > 0.5) are predicted as AMPs.

[c] Sequence similarity to the most similar known AMP sequence from Antimicrobial Peptide Database (APD3, downloaded on July 11, 2022) (Wang et al. 2016) and Database of Anuran Defense Peptides (DADP, downloaded on December 6, 2018) (Novković et al. 2012).

[d] Similarity to the most similar known chicken AMP in predicted 3D structures (i.e., reference chicken AMP) from APD3 (downloaded on October 14, 2022) (Wang et al. 2016). 3D structures of the peptides were predicted by ColabFold (Jumper et al. 2021; Mirdita et al. 2022), and the similarity between two peptides in predicted 3D structures were evaluated by TM-score (Zhang and Skolnick 2004) normalized by the average peptide sequence length using TM-align (Zhang and Skolnick 2005).

[e] The most similar known chicken AMP in predicted 3D structures.

[*] DiDi3 and MuMu2 were not successfully synthesized and were excluded for further tests.

Table S4: Overview of the 40 putative AMP sequences mined from the UniProtKB/Swiss-Prot database that have been prioritized for synthesis regarding their corresponding parent sequence information in the database. This table supplements Table S3 with information about the 40 putative AMP sequences regarding their corresponding parent sequences. The top section shows the 30 most similar putative AMPs to the known chicken AMPs in predicted three-dimensional (3D) structures as measured by TM-scores, while the bottom section shows the 10 least similar ones to the known chicken AMPs in predicted 3D structures.

Putative AMP name	Position in parent sequence[a]	UniProt entry ID	Source organism	Source organism category[b]
DaRe1	17 – 41	B0S8I0	*Danio rerio*	Others
ScPo1	20 – 51	Q9Y7K8	*Schizosaccharomyces pombe*	Others
ArTh10	[29 – 45] + [356 – 369]	Q7Y223	*Arabidopsis thaliana*	Plant
HoSa1	299 – 324	Q8NH93	*Homo sapiens*	Mammal
HoSa2	34 – 55	Q8IYJ2	*Homo sapiens*	Mammal
DiVi1	983 – 1011	Q24702	*Dictyocaulus viviparus*	Others
DiDi2	38 – 67	Q54GV3	*Dictyostelium discoideum*	Others
DiDi3*	145 – 169	Q54UP0	*Dictyostelium discoideum*	Others
LySt2	[187 – 199] + [223 – 225] + [292 – 306]	P19802	*Lymnaea stagnalis*	Others
MuMu1	16 – 50	P09925	*Mus musculus*	Mammal
CaGl1	31 – 60	Q6FWE8	*Candida glabrata*	Others
HoSa3	145 – 166	Q9H3J6	*Homo sapiens*	Mammal
BoMo2	44 – 67	P82003	*Bombyx mori*	Insect
ScPo2	36 – 57	O74430	*Schizosaccharomyces pombe*	Others
DiDi1	539 – 561	Q54TM2	*Dictyostelium discoideum*	Others
RaNo1	194 – 227	P09320	*Rattus norvegicus*	Mammal

HoSa4	39 – 58	A8MTL0	*Homo sapiens*	Mammal
GaGa2	23 – 33	P10039	*Gallus gallus*	Others
MuMu2*	456 – 477	Q2TB54	*Mus musculus*	Mammal
RaNo2	110 – 133	Q5U2T1	*Rattus norvegicus*	Mammal
HoSa5	[23 – 38] + [189 – 197]	O75596	*Homo sapiens*	Mammal
EiHe1	32 – 62	A0A291NUI5	*Eidolon helvum*	Mammal
PlVi1	99 – 117	P0CV63	*Plasmopara viticola*	Others
MuMu3	194 – 227	O35256	*Mus musculus*	Mammal
SuSc1	22 – 44	P53366,<br>O62827	*Sus scrofa*,<br>*Bos taurus*	Mammal
ArTh11	28 – 49	Q6NKN8	*Arabidopsis thaliana*	Plant
MoIn1	1861 – 1879	Q09WW0	*Morus indica*	Plant
HoSa6	23 – 33	P24821,<br>Q29116	*Homo sapiens*,<br>*Sus scrofa*	Mammal
CaGl2	29 – 48	P05040	*Candida glabrata*	Others
HoSa7	75 – 109	Q99678	*Homo sapiens*	Mammal
GaGa4	[25 – 29] + [286 – 300]	Q92080	*Gallus gallus*	Others
OrSa6	25 – 36	Q5VRI5	*Oryza sativa subsp. japonica*	Plant
GoGo1	744 – 765	A1YF22,<br>A1YG99,<br>A2T7S4,<br>A2T771,<br>Q9UKY1	*Gorilla gorilla gorilla*,<br>*Pan paniscus*,<br>*Pongo pygmaeus*,<br>*Pan troglodytes*,<br>*Homo sapiens*	Mammal
HoSa8	200 – 205	Q9BXP8	*Homo sapiens*	Mammal
HoSa9	562 – 584	Q68DV7	*Homo sapiens*	Mammal

DrMe6	17 – 25	O46201	*Drosophila melanogaster*	Insect
MuMu4	469 – 485	Q9Z0L3	*Mus musculus*	Mammal
HoSa10	286 – 292	Q86YB7	*Homo sapiens*	Mammal
UnBi1	10 – 17	C0HKK6	*Unedogemmula bisaya*	Others
ScPo3	24 – 29	O42663	*Schizosaccharomyces pombe*	Others

[a] Position of the putative mature AMP sequence in the corresponding parent sequence. For a putative AMP sequence that is the recombination of multiple cleaved peptide sequences, the positions of those cleaved peptide sequences are presented in brackets with plus signs connecting them to each other.

[b] Source organisms are classified into five categories: amphibian, plant, insect, mammal, and others.

[*] DiDi3 and MuMu2 were not successfully synthesized and were excluded for further tests.

Table S5: Seven reference chicken AMP sequences for the 40 putative AMPs mined from the UniProtKB/Swiss-Prot database and prioritized for synthesis. The AMP sequences listed in this table are the seven reference chicken AMPs for the 40 putative AMPs listed in Table S3. Only the three reference chicken AMPs for the top 30 putative AMPs which share highest similarities to known chicken AMPs in predicted three-dimensional (3D) structures were prioritized for synthesis and further tests (i.e., Chicken CATH-2, Chicken CATH-3, and Ovipin).

APD3 ID	Peptide name	Sequence	# aa	Net charge[a]	Molecular weight (Da)
AP00548	Chicken CATH-2 (van Dijk et al. 2005)	RFGRFLRKIRRFRPKVTITIQGS ARFG	27	9	3264.92
AP00613	Chicken CATH-3 (Xiao et al. 2006)	RVKRFWPLVPVAINTVAAGINL YKAIRRK	29	7	3351.09
AP03457	Ovipin (dos Santos et al. 2022)	YVSPVAIVKGLNIPL	15	1	1582.95
AP03014	OvoDBβ (Yu et al. 2018)	QSKKCCGRCSSRMCTKREKEE HTEDCRGSFCCLTHRKKK	39	7	4609.37
AP02878	CHB1 (Vasilchenko et al. 2016)	VLSAADKNNVKGIFTKIAGHA EEYGAETLERMFTTYPPTKTY	42	0	4664.27
AP02879	CHB2 (Vasilchenko et al. 2016)	LTAEDKKLIQQAWEKAASHQE EFGAEALTRMFTTYPQTKTY	41	-1	4762.33
AP00265	Chicken Heterophil Peptide 2 (Evans et al. 1994)	GRKSDCFRKNGFCAFLKCPYL TLISGLCSFHLC	33	4	3729.47

[a] Net charge at pH = 7.

Table S6: Antimicrobial susceptibility testing and hemolysis experiment results of the 38 successfully synthesized putative AMPs mined from the UniProtKB/Swiss-Prot database and tested *in vitro*. Peptides were tested for their antimicrobial activity against *Escherichia coli* ATCC 25922 and *Staphylococcus aureus* ATCC 29213 for their minimum inhibitory concentration (MIC) and minimum bactericidal concentration (MBC) values. Porcine red blood cells (RBCs) were used to test the hemolytic activity of the selected peptides for their hemolytic concentration (HC_{50}) values. Data is presented as the lowest effective peptide concentration range (µg/mL) observed in three independent experiments performed in duplicate, with one maximum data point and one minimum data point dropped for each measurement. The top section shows results of the 28 successfully synthesized peptides among the top 30 peptides in similarities to known chicken AMPs in predicted three-dimensional (3D) structures as measured by TM-scores, while the second section shows results of the 10 least similar ones to known chicken AMPs in predicted 3D structures. Results of the three reference chicken AMPs (Chicken CATH-2 (van Dijk et al. 2005), Chicken CATH-3 (Xiao et al. 2006), and Ovipin (dos Santos et al. 2022)) for the top 30 peptides are listed in the third section for comparison. The control peptides in the bottom section includes: a positive control peptide Ranatuerin-4 (Goraya et al. 1998) and a negative control peptide OT15.

**Peptide name**	**Antimicrobial susceptibility testing**				**Hemolysis testing**[a]
	***E. coli* ATCC 25922**		***S. aureus* ATCC 29213**		**Porcine RBCs**
	**MIC (µg/mL)**	**MBC (µg/mL)**	**MIC (µg/mL)**	**MBC (µg/mL)**	**HC_{50} (µg/mL)**
DaRe1	32 – 64	32 – 64	>128	>128	>128
ScPo1	>128	>128	>128	>128	—
ArTh10	>128	>128	>128	>128	—
HoSa1	16	16 – 32	128	—*	>128
HoSa2	>128	>128	>128	>128	—
DiVi1	>128	>128	>128	>128	—
DiDi2	16	16	>128	>128	>128
LySt2	>128	>128	>128	>128	—
MuMu1	≥128	—*	>128	>128	—
CaGl1	>128	>128	>128	>128	—

HoSa3	>128	>128	>128	>128	—
BoMo2	>128	>128	>128	>128	—
ScPo2	>128	>128	>128	>128	—
DiDi1	>128	>128	>128	>128	—
RaNo1	>128	>128	>128	>128	—
HoSa4	32	32	64 – 128	—*	>128
GaGa2	128	—*	>128	>128	>128
RaNo2	>128	>128	>128	>128	—
HoSa5	>128	>128	>128	>128	—
EiHe1	>128	>128	>128	>128	—
PlVi1	4 – 8	8	32	32	>128
MuMu3	>128	>128	>128	>128	—
SuSc1	128	—*	>128	>128	>128
ArTh11	64 – 128	—*	>128	>128	>128
MoIn1	64 – >128	—*	>128	>128	—
HoSa6	>128	>128	>128	>128	—
CaGl2	>128	>128	>128	>128	—
HoSa7	64 – >128	—*	>128	>128	—
GaGa4	>128	>128	>128	>128	—
OrSa6	>128	>128	>128	>128	—
GoGo1	64	—*	>128	>128	>128
HoSa8	>128	>128	>128	>128	—
HoSa9	>128	>128	>128	>128	—
DrMe6	>128	>128	>128	>128	—
MuMu4	>128	>128	>128	>128	—

HoSa10	>128	>128	>128	>128	—
UnBi1	128	—*	>128	>128	>128
ScPo3	>128	>128	>128	>128	>128
Chicken CATH-2	8 – 16	8 – 16	32	32	>128
Chicken CATH-3	2 – 4	2 – 4	2	2 – 4	>128
Ovipin	>128	>128	>128	>128	>128
Ranatuerin-4	4	4	1 – 2	2	16
OT15[b]	>128	>128	>128	>128	>128

[a] Hemolysis experiments were not performed for putative AMPs that did not show any antimicrobial activity (MIC > 128 μg/mL) in at least two repeats for each bacterial strain tested.

[b] OT15 (TKPKGTKPKGTKPKG) is a truncated form of a negative control peptide OT20 (Horváti et al. 2017) used in previous studies.

* MBC values were not tested for experiments revealing antimicrobial activity but with high MIC values of ≥ 64 μg/mL.

'—' = not tested.

References

van Dijk A, Veldhuizen EJA, van Asten AJAM, Haagsman HP. 2005. CMAP27, a novel chicken cathelicidin-like antimicrobial protein. Vet Immunol Immunopathol. 106(3–4):321–327. doi:10.1016/j.vetimm.2005.03.003.

Duckert P, Brunak S, Blom N. 2004. Prediction of proprotein convertase cleavage sites. Protein Eng Des Sel. 17(1):107–112. doi:10.1093/protein/gzh013.

Evans EW, Beach GG, Wunderlich J, Harmon BG. 1994. Isolation of antimicrobial peptides from avian heterophils. J Leukoc Biol. 56(5):661–665. doi:10.1002/jlb.56.5.661.

Goraya J, Knoop FC, Conlon JM. 1998. Ranatuerins: Antimicrobial Peptides Isolated from the Skin of the American Bullfrog,Rana catesbeiana. Biochem Biophys Res Commun. 250(3):589–592. doi:10.1006/bbrc.1998.9362.

Horváti K, Bacsa B, Mlinkó T, Szabó N, Hudecz F, Zsila F, Bősze S. 2017. Comparative analysis of internalisation, haemolytic, cytotoxic and antibacterial effect of membrane-active cationic peptides: aspects of experimental setup. Amino Acids. 49(6):1053–1067. doi:10.1007/s00726-017-2402-9.

Jumper J, Evans R, Pritzel A, Green T, Figurnov M, Ronneberger O, Tunyasuvunakool K, Bates R, Žídek A, Potapenko A, et al. 2021. Highly accurate protein structure prediction with AlphaFold. Nature. 596(7873):583–589. doi:10.1038/s41586-021-03819-2.

Li C, Sutherland D, Hammond SA, Yang C, Taho F, Bergman L, Houston S, Warren RL, Wong T, Hoang LMN, et al. 2022. AMPlify: attentive deep learning model for discovery of novel antimicrobial peptides effective against WHO priority pathogens. BMC Genomics. 23:77. doi:10.1186/s12864-022-08310-4.

Li C, Warren RL, Birol I. 2023. Models and data of AMPlify: a deep learning tool for antimicrobial peptide prediction. BMC Res Notes. 16:11. doi:10.1186/s13104-023-06279-1.

Lin D, Sutherland D, Aninta SI, Louie N, Nip KM, Li C, Yanai A, Coombe L, Warren RL, Helbing CC, et al. 2022. Mining Amphibian and Insect Transcriptomes for Antimicrobial Peptide Sequences with rAMPage. Antibiotics. 11(7):952. doi:10.3390/antibiotics11070952.

Meher PK, Sahu TK, Saini V, Rao AR. 2017. Predicting antimicrobial peptides with improved accuracy by incorporating the compositional, physico-chemical and structural features into Chou's general PseAAC. Sci Rep. 7:42362. doi:10.1038/srep42362.

Mirdita M, Schütze K, Moriwaki Y, Heo L, Ovchinnikov S, Steinegger M. 2022. ColabFold: making protein folding accessible to all. Nat Methods. 19(6):679–682. doi:10.1038/s41592-022-01488-1.

Novković M, Simunić J, Bojović V, Tossi A, Juretić D. 2012. DADP: the database of anuran defense peptides. Bioinformatics. 28(10):1406–1407. doi:10.1093/bioinformatics/bts141.

dos Santos SR, Miranda A, da Silva Junior PI. 2022. Ovipin: a new antimicrobial peptide from chicken eggs Gallus gallus. bioRxiv.

doi:10.1101/2021.09.28.462162. https://www.biorxiv.org/content/10.1101/2021.09.28.462162v2.

Vasilchenko AS, Rogozhin EA, Vasilchenko AV, Kartashova OL, Sycheva MV. 2016. Novel haemoglobin-derived antimicrobial peptides from chicken (Gallus gallus) blood: purification, structural aspects and biological activity. J Appl Microbiol. 121(6):1546–1557. doi:10.1111/jam.13286.

Veltri D, Kamath U, Shehu A. 2018. Deep learning improves antimicrobial peptide recognition. Bioinformatics. 34(16):2740–2747. doi:10.1093/bioinformatics/bty179.

Wang G, Li X, Wang Z. 2016. APD3: the antimicrobial peptide database as a tool for research and education. Nucleic Acids Res. 44(D1):D1087–D1093. doi:10.1093/nar/gkv1278.

Xiao X, Wang P, Lin W-Z, Jia J-H, Chou K-C. 2013. iAMP-2L: A two-level multi-label classifier for identifying antimicrobial peptides and their functional types. Anal Biochem. 436(2):168–177. doi:10.1016/j.ab.2013.01.019.

Xiao Y, Cai Y, Bommineni YR, Fernando SC, Prakash O, Gilliland SE, Zhang G. 2006. Identification and Functional Characterization of Three Chicken Cathelicidins with Potent Antimicrobial Activity. J Biol Chem. 281(5):2858–2867. doi:10.1074/jbc.M507180200.

Yu L-T, Xiao Y-P, Li J-J, Ran J-S, Yin L-Q, Liu Y-P, Zhang L. 2018. Molecular characterization of a novel ovodefensin gene in chickens. Gene. 678:233–240. doi:10.1016/j.gene.2018.08.029.

Zhang Y, Skolnick J. 2004. Scoring function for automated assessment of protein structure template quality. Proteins. 57(4):702–710. doi:10.1002/prot.20264.

Zhang Y, Skolnick J. 2005. TM-align: a protein structure alignment algorithm based on the TM-score. Nucleic Acids Res. 33(7):2302–2309. doi:10.1093/nar/gki524.
